# Seasonal and Genotypic Changes in *Escherichia coli* Phylogenetic Groups in the Yeongsan River Basin of South Korea

**DOI:** 10.1371/journal.pone.0100585

**Published:** 2014-07-07

**Authors:** Jeonghwan Jang, Doris Y. W. Di, Anna Lee, Tatsuya Unno, Michael J. Sadowsky, Hor-Gil Hur

**Affiliations:** 1 School of Environmental Science and Engineering, Gwangju Institute of Science and Technology, Gwangju, Republic of Korea; 2 International Environmental Analysis and Education Center, Gwangju Institute of Science and Technology, Gwangju, Republic of Korea; 3 College of Molecular Life Sciences, Jeju National University, Jeju, Republic of Korea; 4 Department of Soil, Water and Climate, University of Minnesota, St. Paul, Minnesota, United States of America; 5 BioTechnology Institute, University of Minnesota, St. Paul, Minnesota, United States of America; U. S. Salinity Lab, United States of America

## Abstract

With 3,480 *E. coli* strains isolated from the Yeongsan River basin, South Korea, correlations between phylogenetic groups and horizontal fluorophore enhanced rep-PCR (HFERP) genotypes were examined, and environmental factors affecting *E. coli* phylogenetic groups in the river water were determined. Interestingly, multidimentional scaling (MDS) analyses based on HFERP DNA fingerprint data indicated that *E. coli* in phylogenetic groups A and B1 were uniquely clustered. Results of self-organized maps (SOMs) analyses also indicated that *E. coli* phylogenetic groups were seasonally affected by water temperature, with greater occurrences of phylogenetic groups A and B1 in low and high temperature seasons, respectively. The presence of *E. coli* in phylogenetic groups A and B1 were inversely related. Furthermore, redundancy analysis (RDA) revealed that phylogenetic group B1 correlated positively with temperature, strain diversity, and biochemical oxygen demand (BOD) but negatively with phylogenetic group A. Results of this study indicated that while *E. coli* strains could be clustered based on their genotypes and environment conditions, their phylogenetic groups did not change in relation to the same conditions. The distributional differences of phylogenetic groups among *E. coli* populations in different environments may be caused by different genomic adaptability and plasticity of *E. coli* strains belonging to each phylogenetic group. Although several previous studies have reported different *E. coli* ecological structures depending on their origins, this study is a first description of the specific environmental factors affecting *E. coli* phylogenetic groups in river water.

## Introduction

*Escherichia coli* is generally considered to be a commensal bacterium found in the intestinal tracts of warm-blooded animals. Although this bacterium has been adopted as one of the best indicator organisms to examine fecal pollution in freshwater environments [1], its effectiveness as a fecal indicator has been questioned since several recent studies have reported naturalized *E. coli* populations which can survive for long period of time and reproduce in the environments outside the host bodies such as sand, soil and sediments [2]–[5]. Surrounding environmental conditions have been suggested to influence the genotypic traits of *E. coli* in the extraintestinal environments [6]. In a previous study, it was observed that genotypic richness of *E. coli* strains obtained from surface water of the Yeongsan River of South Korea was fluctuated with environmental conditions, especially water temperature, and season-specific *E. coli* genotypes were found in cold months [7]. Taken together, in order to fully understand ecology of this bacterium and its use as a fecal indicator, ecological approaches of *E. coli* community dynamics in the environments seem to be essential.

*E. coli* strains can be mainly assigned to four major phylogenetic groups: A, B1, B2 and D [8]. Recently, the extensive multi-locus sequence typing (MLST) and genome data sets for *E. coli* strains have refined our knowledge of *E. coli* phylogenetic group structure, and new phylogenetic groups of *E. coli* are now recognized such as phylogenetic groups C, E, F, and *Escherichia* clade I, which are comparatively rare to the major phylogenetic groups [9]–[14].

Strains belonging to each phylogenetic group possess different phenotypic and genotypic traits relative to each other [15], [16]. Furthermore, the four major phylogenetic groups have been reported to differ in their ecological habitats. While phylogenetic groups B2 and D have been less frequently found than A or B1 in the environment [17], strains in group B2 have been observed to persist longer than the other groups in infants [18]. Moreover, *E. coli* strains in phylogenetic group B2 or D were more frequently isolated from extraintestinal sites within host bodies than group A or B1 strains [16]. Some strains belonging to phylogenetic group B1 were reported to persist in water environments [17], [19]. Many studies have also reported the relationship between virulence and phylogenetic groups of *E. coli*. Phylogenetic group B2 strains tend to be more virulent than other groups [20]–[23], and virulence genes were most frequently present in phylogenetic group B1 strains in the absence of B2 strains [24]. Thus, phylogenetic group identification of unknown *E. coli* strains may provide important information of their physiological and ecological aspects.

While initial studies used multilocus enzyme electrophoresis (MLEE) to classify *E. coli* strains into phylogenetic groups [25], Clermont and colleagues (2000) developed a multiplex PCR-based method to determine the four major phylogenetic groups of *E. coli* strains [26]. This method was further validated by Gordon et al. (2008) and showed that the PCR-based and multi-locus sequence typing (MLST) methods classified 80–85% of 662 *E. coli* isolates into the same phylogenetic groups [27]. These methods have been often used for the phylogenetic grouping of *E. coli* strains from various sources [22], [24], [28], [29] since it reduces the time and cost of experimental procedures needed to examine a large number of strains. Recently, a new quadruplex PCR method enables an *E. coli* strain to be assigned to one of the seven phylogenetic groups (A, B1, B2, C, D, E, and F) and *Escherichia* clade I, which is also considered to be a phylogenetic group of *E. coli* [30].

The horizontal fluorophore-enhanced rep-PCR (HFERP) DNA fingerprinting technique enables accurate genotyping of *E. coli* strains [31], [32]. However, no direct correlation between HFERP DNA fingerprint patterns and phylogenetic groups of *E. coli* strains was described previously [24], [31]. Nevertheless, since rep-PCR DNA fingerprint patterns are susceptible to alteration of microbial genome structure, the technique can be used to study plasticity, molecular phylogeny, and evolution of microbial genomes [31]. The HFERP DNA fingerprinting technique, which revealed 1749 genotypes among 3480 *E. coli* isolates in a previous study [7], provides the much higher discriminatory power than the MLST or multiplex PCR methods assigning a *E. coli* strain to one of the eight phylogenetic groups as described above.

The aim of this study was to: 1) examine the relationship between phylogenetic groups and the HFERP DNA fingerprints of 3,480 *E. coli* strains isolated from the Yeongsan River basin, 2) determine correlations between *E. coli* phylogenetic groups and environmental factors in the environment, and 3) relations between virulence gene profiles of shiga toxigenic *E. coli* (STEC) and the phylogenetic groups of *E. coli* in the environment.

## Materials and Methods

### Environmental samples and bacterial strains

The sampling approach used and the isolation of *E. coli* strains from surface water samples was done as described previously [7]. A total of 3,480 *E. coli* strains were the same as those used in a previous study [7], and were subjected to further experiments here. Briefly, surface water samples were collected monthly from the 7 sites of the Yeongsan River basin (Table 1), including 3 agricultural sites, 3 urban sites, and 1 site affected by agricultural and urban, from April to December 2009, then sixty *E. coli* strains were isolated from each water sample but samples less than 60 isolates were excluded from further analyses. The membrane filtration technique with mTEC agar (Difco, Detroit, MI) was employed to obtain *E. coli* strains from freshwater samples [1]. ChromAgar ECC (Chromagar Microbiology, Paris, France) was used to reconfirm the phenotypical identity of potential *E. coli* isolates [6]. As practical point of view considering the large number of *E. coli* isolates, no further confirm tests for *E. coli* identification were proceeded since 97.3% of presumptive *E. coli* colonies obtained from the membrane filtration method with mTEC agar medium have been identified as *E. coli* strains in a previous study [33]. All *E. coli* isolates were preserved in LB freezing medium at −70°C [34], and their genomic DNA was prepared by boiling in 0.05 N NaOH. Colonies formed on LB agar plates were suspended in 100 µl of 0.05 N NaOH. The cells were lysed at 95°C for 15 min, and cell debris was precipitated by brief centrifugation. A dilution (1∶10) of the supernatant was used as a DNA template of the further PCR assays.

**Table 1:** Information of each sampling location.

Sampling sites	Sub-basin	Major land use affecting sub-basin	Latitude and longitude
YS1	Manbong tributary	Agricultural	34°59'39.66"N, 126°42'5.92"E
YS2	Jangseong tributary	Agricultural	35° 3'10.05"N, 126°44'29.45"E
YS3	Main stream of the Yeongsan River	Agricultural and Urban	35° 6'34.02"N, 126°49'8.87"E
YS4	Orye tributary	Agricultural	35°16'57.44"N, 126°57'29.88"E
GJ1	Gwangju tributary	Urban	35° 9'11.54"N, 126°50'3.90"E
GJ2	Gwangju tributary	Urban	35°10'7.19"N, 126°53'3.13"E
GJ3	Gwangju tributary	Urban	35° 7'45.20"N, 126°55'41.71"E

Moreover, water quality parameters such as temperature, pH, biochemical oxygen demand (BOD), and *E. coli* MPN values were also obtained from all water samples by using the YSI 6600 Sonde (YSI Incorporated, Yellow Springs, OH) and the Colilert (IDEXX Laboratories, Inc., Westbrook, MN) system as described previously [7].

All the sampling sites were located in the Yeongsan River basin which is one of public rivers managed by Korean government. While the Youngsanriver Environmental Management Office is the government organization which manages the study area, no specific permission was required for any research activities performed in this study.

#### Horizontal fluorophore-enhanced rep-PCR (HFERP) DNA fingerprinting technique and defining HFERP genotypes of *E. coli* strains

Horizontal fluorophore-enhanced rep-PCR (HFERP) genotyping of the *E. coli* strains used in this study was done previously as described in our previous study [7]. Briefly, the BOX A1R (5′-CTACGGCAAGGCGACGCTGACG-3′) primer labeled with 6-FAM (6-carboxyfluorescein; Genotech Co. Ltd., Korea) was employed for the rep-PCR genotyping, then amplified DNA fragments and the Genescan-2500 ROX (6-carboxy-X-rhodamine) (Applied Biosystems, Foster City, CA) internal standard were separated by gel electrophoresis (70 V for 16 hours at 4°C) in 0.5 x TAE buffer using 1% Seachem LE agarose gels (FMC Bioproducts, Rockland, ME). Gel images were obtained by using by using a Typhoon 9400 variable mode imager (Molecular Dynamics/Amersham Biosciences, Sunnyvale, CA), and the image files were analyzed using BioNumerics v.6.01 software (Applied Maths, Sint-Martens-Latem, Belgium). Detailed protocols for HFERP DNA fingerprinting are accessible at http://www.ecolirep.umn.edu.

#### Phylogenetic grouping and virulence detection of *E. coli* strains

The phylogenetic grouping of the 3,480 *E. coli* strains was done using the Clermont multiplex PCR methods as described previously [26]. Briefly, the multiplex PCR assay was performed in 20 µl total volume containing 2 µl of 10X PCR buffer supplied with Top DNA polymerase (Bioneer, Korea), 1.5 µl of 20 mM MgCl_2_, 20 pmol of each primer shown in top part of Table 2, 2 µM of each deoxynucleoside triphosphate, 2.5 U of Top DNA polymerase (Bioneer, Korea), and 3 µl of bacterial lysate. The PCR steps were composed of denaturation for 4 min at 94°C, 30 cycles at 94°C for 5 s and 10 s at 59°C, and a final extension step of 5 min at 72°C.

**Table 2:** PCR primers used for phylogenetic grouping and virulence gene detection among *E. coli* strains.

Target genes or DNA fragment	Primer sequence	Amplicon size (bp)	Reference
*chuA*	5′-GAC GAA CCA ACG GTC AGG AT-3′	279	[26]
	5′-TGC CGC CAG TAC CAA AGA CA-3′		
*yjaA*	5′-TGA AGT GTC AGG AGA CGC TG-3′	211	
	5′-ATG GAG AAT GCG TTC CTC AAC-3′		
TspE4.C2	5′-ATG GAG AAT GCG TTC CTC AAC-3′	152	
	5′-GAG TAA TGT CGG GGC ATT CA-3′		
*stx_1_*	5′-ATA AAT CGC CAT TCG TTG ACT AC-3′	180	[35]
	5′-AGA ACG CCC ACT GAG ATC ATC-3′		
*stx_2_*	5′-GGC ACT GTC TGA AAC TGC TCC-3′	255	
	5′-TCG CCA GTT ATC TGA CAT TCT G-3′		
*eaeA*	5′-GAC CCG GCA CAA GCA TAA GC-3′	384	
	5′-CCA CCT GCA GCA ACA AGA GG-3′		
*hlyA*	5′-GCA TCA TCA AGC GTA CGT TCC-3′	534	
	5′-AAT GAG CCA AGC TGG TTA AGC T-3′		

The presence of virulence genes among the *E. coli* strains related to shiga toxin producing *E. coli* (STEC), enterohemorrhagic *E. coli* (EHEC), and enteropathogenic *E. coli* (EPEC) (*eaeA*, *stx_1_*, and *stx_2_*) was determined by using multiplex PCR as previously described [35]. Briefly, the target genes were amplified in 25 µl reaction mixture containing 200 µM concentrations of deoxynuceloside triphosphates, 250 nM concentration of each primer shown in bottom part of Table 2, 1 U of Top DNA polymerase (Bioneer, Korea), 2.5 µl of 10X PCR buffer supplied with Top DNA polymerase (Bioneer, Korea), 2 mM MgCl_2_, and 2 µl of bacterial lysate. The reaction mixtures were subjected to first 10 cycles of 1 min of denaturation at 95°C, 2 min of annealing at 65°C, and 1.5 min of elongation at 72°C; a second 15 cycles of 1 min of denaturation at 95°C, 2 min of annealing at 60°C, and 1.5 min of elongation at 72°C; and a third 10 cycles of 1 min of denaturation at 95°C, 2 min of annealing at 65°C, and 2.5 min of elongation at 72°C. All of the PCR products were assessed by using an automated capillary electrophoresis system, QIAxcel (Qiagen, Hilden, Germany).

### Statistical methods

Multidimentional scaling (MDS) analyses were done using BioNumerics v.6.01 software (Applied Maths, Sint-Martens-Latem, Belgium) and provided a three dimentional representation of *E. coli* phylogenetic groups based on similarities between HFERP DNA fingerprints. The correct assignment of *E. coli* strains to phylogenetic group was evaluated by using Jackknife analysis, with maximum similarities [32]. Self-organizing maps (SOMs) analyses using the SOM Toolbox implements (Laboratory of Computer and Information Science, Helsinki, Norway) of Matlab 2009b (MathWorks, Natick, MA) were used to determine if there were correlations between *E. coli* phylogenetic groups and environmental factors, including temperature, pH, BOD, and *E. coli* MPN values. Raw data for the SOMs analyses are organized as presented in Table S1 (supplementary file). The occurrence of four *E. coli* phylogenetic groups and the five water quality parameters described previously [7] were used as input variables for the SOMs analyses. The visualized map images, which were automatically optimized at 42 SOM cells, were generated with minimum quantization error (1.58) and the least topographic errors (0.034). Quantization error (QE) and topographic error (TE) were estimated to confirm reliable resolution and topology conservation of the SOMs analyses, and these are meanings of the statistical significance. QE is the average distance between each data vector and its best matching unit (BMU), measures map resolution. TE measures map quality, which represents the proportion of all data vectors for which 1st and 2nd BMUs are not adjacent, and is thus used for the measurement of topology preservation. The results of the SOMs analyses are based on statistical procedures, and map size and structure (n×m) are automatically optimized and determined based on the least QE and TE values among numerous cases. Borders between clusters existing in the map were defined by using a hierarchical cluster analysis. Features and analytic procedures of SOMs analyses have been described previously [7], [36]–[41].

Redundancy analysis (RDA), one of multivariate analyses included in software packages CANOCO v5.0 (Microcomputer Power, Ithaca, New York), was performed to test which environmental factors significantly correlated with the variation in the *E. coli* phylogenetic groups [42]. Five parameters including temperature, pH, BOD, *E. coli* MPN values, and strain diversity were used for the explanatory variables constraining the occurrences of the *E. coli* phylogenetic groups A and B1, which occupied over 80% of total *E. coli* strains tested in this study. Response data in the analysis have a gradient 0.8 standard deviation (SD) units, suggesting that *E. coli* phylogenetic group response to the gradient of the environmental factors is linear. The forward selection method was used to determine significant environmental variables affecting the *E. coli* phylogenetic groups using the 999 Monte Carlo permutations at P<0.05 [43].

## Results

### Genotypic separation of *E. coli* strains in different phylogenetic groups

**Figure 1:**
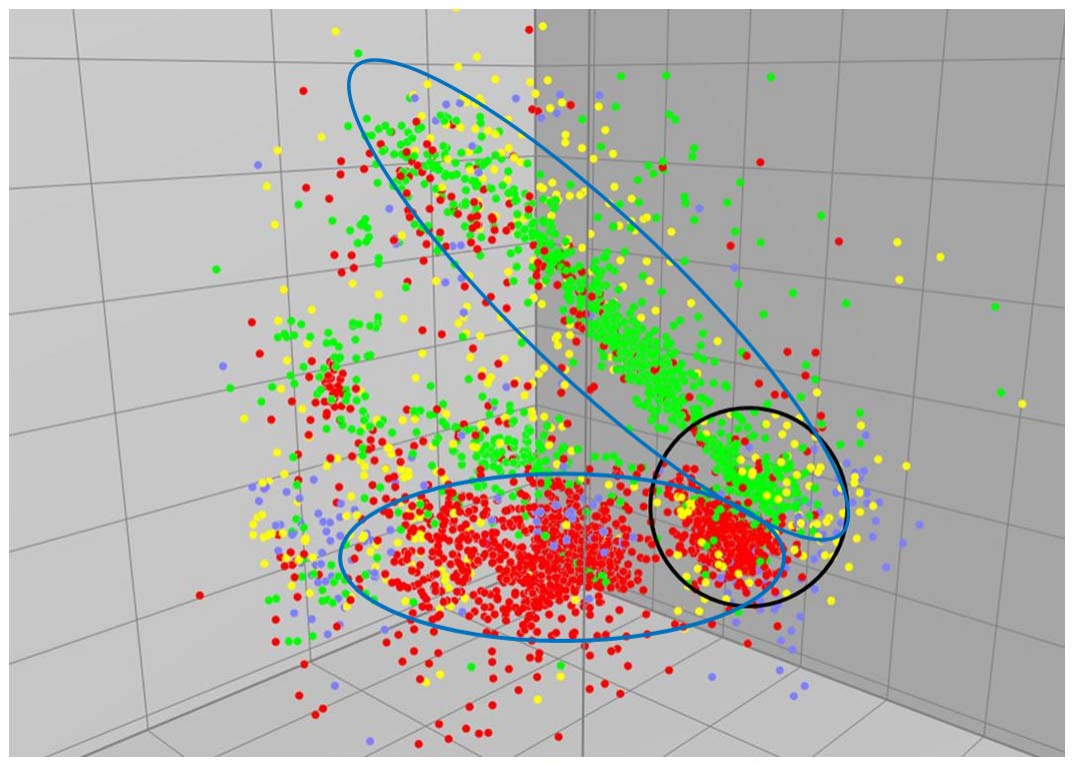
Multidimensional scaling (MDS) analyses of *E. coli* HFERP DNA fingerprints given colors according to their phylogenetic groups. Legend: A (red), B1 (green), B2 (sky-blue), and D (yellow).

The 3,480 *E. coli* strains isolated from the Yeongsan River basin in Korea were comprised of 1,724 (49.5%), 1,192 (34.3%), 188 (5.4%), and 376 (10.8%) strains in phylogenetic groups A, B1, B2, and D, respectively. The MDS analyses were performed to investigate a correlation between HFERP DNA fingerprints and phylogenetic groups of *E. coli* strains (Fig. 1). As shown in Figure 1, each dot in the three dimensional space indicates HFERP DNA fingerprint of each *E. coli* strain, and it was given a color according to one of the phylogenetic groups the *E. coli* strain belongs to. Distances between dots simply reflect similarities between HFERP DNA fingerprints of *E. coli* strains. Interestingly, phylogenetic groups A and B1 showed a tendency to cluster along with genotype distributions (indicated by blue circles). Moreover, the both phylogenetic groups were separated even within the tightly clustered genotypic group (indicated by black circle) obtained from the cold months as described previously [7]. In contrast, phylogenetic groups B2 and D showed no significant clustering along with genotypic distributions in the MDS analyses. As shown in Table 3, the clustering trend of the MDS analyses was reconfirmed by using Jackknife analysis, in which strains were removed from the group one at a time and treated as unknowns for classification. Phylogenetic groups A and B1 showed significantly higher percentages of *E. coli* strains correctly assigned to their own group (90.1% and 89.4%) than phylogenetic groups B2 and D (65.4% and 77.7%) (p<0.05, two-sample *t*-test assuming equal variances using Microsoft Excel).

**Table 3:** The percentage of *E. coli* strains assigned to each phylogenetic group calculated by Jackknife analysis based on HFERP DNA fingerprints data.

Assigned phylgogenetic group	% *E. coli* strains assigned to each group			
	A	B1	B2	D
A	90.1	8.1	8.5	5.9
B1	6.8	89.4	18.1	11.7
B2	1.4	1.3	65.4	4.8
D	1.7	1.2	8.0	77.7

### SOMs analyses for trends of *E. coli* phylogenetic groups

**Figure 2:**
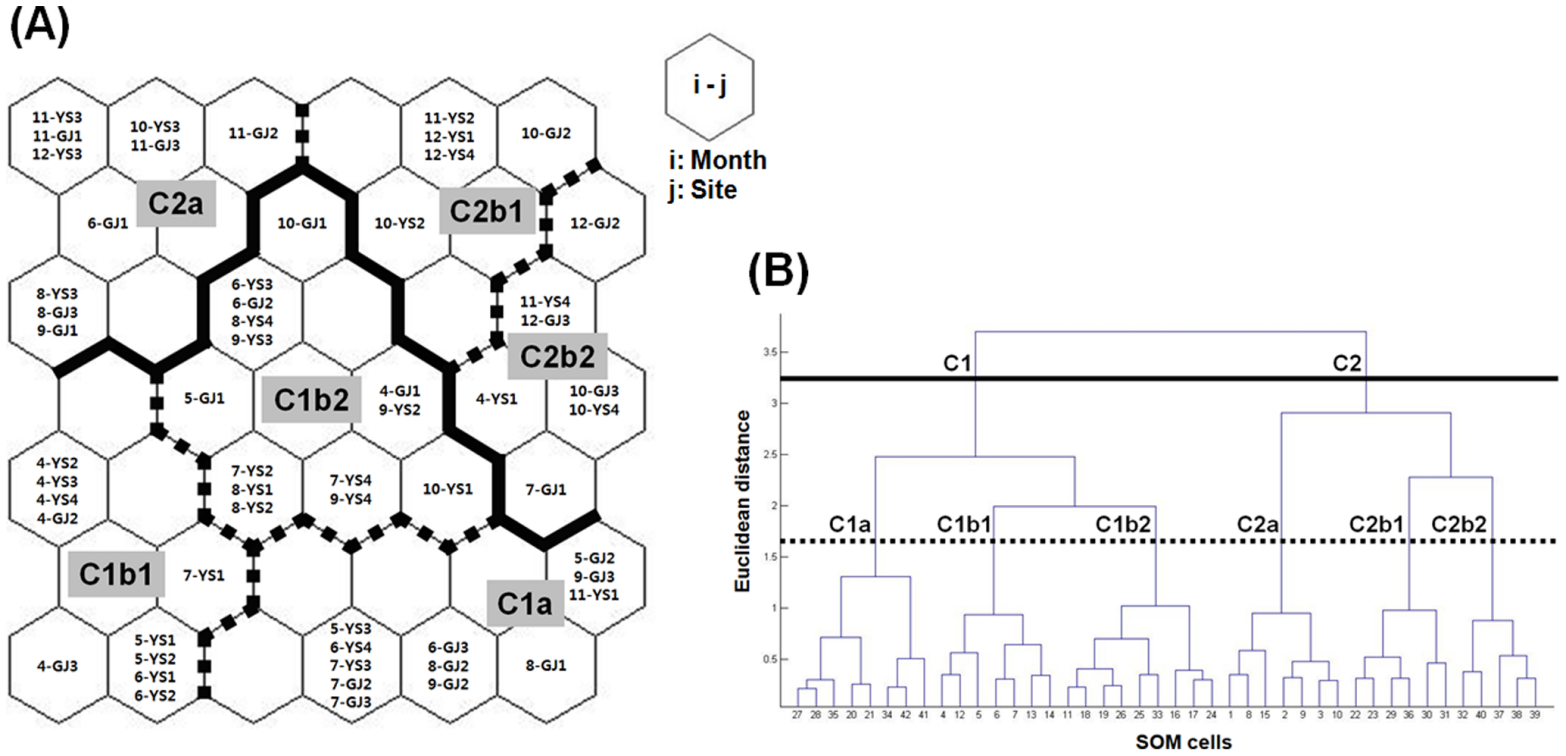
SOMs analyses classification and dendrogram. (A) Six clusters were determined by the SOMs analyses: two main clusters (C1 and C2) including six sub-clusters (C1a, C1b1, C1b2, C2a, C2b1 and C2b2), and (B) dendrogram created by clustering input variables based on the similarity among SOM cells.

**Figure 3:**
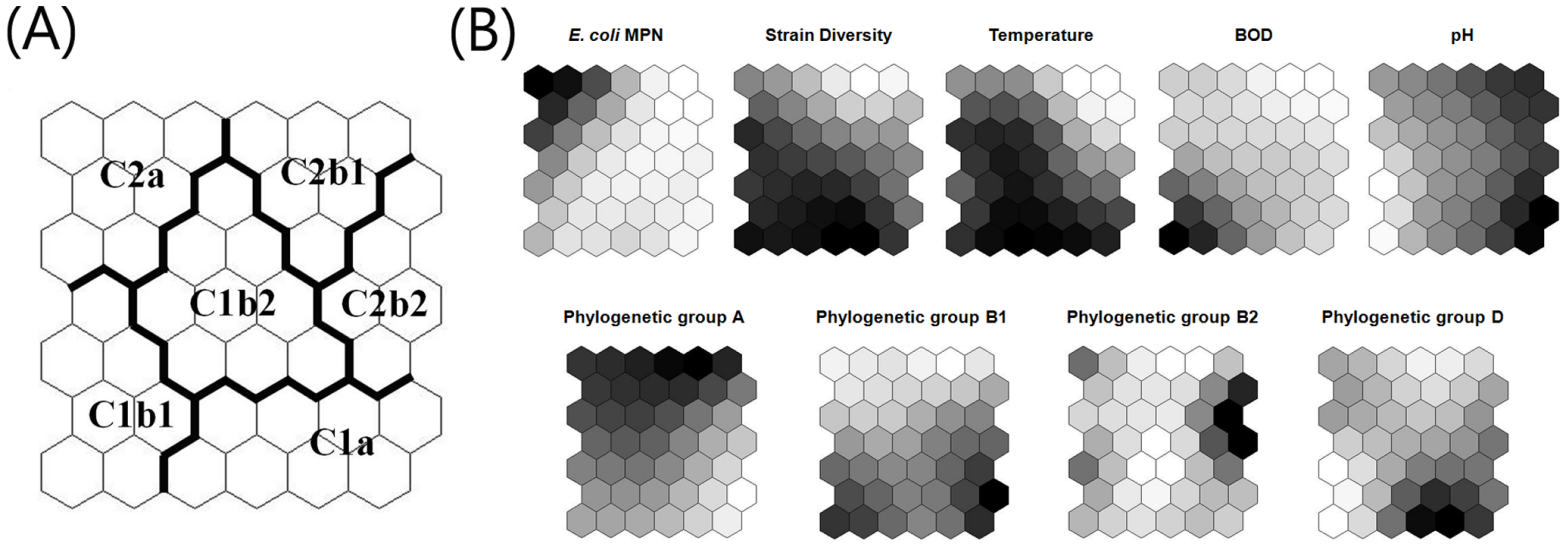
Color maps displaying the intensity of each input variables on the classified SOM cells. Dark and light colors indicate high and low values of each variable, respectively. The intensity of strain diversity was defined as percentage of number of *E. coli* genotypes for total isolates.

Surface water samples were classified into self-organized map (SOM) cells based on their water quality parameters and prevalence of *E. coli* phylogenetic groups. These are used as input variables for the SOMs analyses, and water samples were assigned into SOM cells depending on the similarity among their variables (Fig. 2A). A hierarchical cluster analysis (Fig. 2B) revealed that clusters existed in the map. According to the similarities of the variables in the output cells, clusters were defined as combined cell units of the map (Fig. 2A). As shown in Figure 2, two main clusters (C1 and C2) were further subdivided into six sub-clusters (C1a, C1b1, C1b2, C2a, C2b1, and C2b2). Sub-clusters C2b1 and C2b2 in Cluster C2 were mainly comprised of *E. coli* obtained surface water samples collected from the cold months, October, November, and December. This suggested season-specificity of SOMs. Site-specificity was also observed in sub-cluster C2a since it includes only isolates from surface water samples collected from the urban-affected locations (sites GJ1, GJ2, GJ3, and YS3). Results in Figure 3A and 3B show the contribution of each input variable to the classification of SOMs cells by displaying the distribution of the variable intensities on the map. The intensities are expressed by colors between white and black, with the units of each variable. Dark and light colors indicate high and low values of each variable, respectively. The divisions of SOMs cells shown in Figure 3A can be explained by classification factors which are input variables showing intensity maps in Figure 3B. For instance, a sub-cluster C2a was mainly defined by *E. coli* MPN showing high values of the variable in the cluster. By the same token, a sub-cluster C2b2 was determined by phylogenetic group B2. Furthermore, these results indicated that the relationships between input variables can be observed by comparing the intensity maps to each other. Based on the analyses, the following interpretation could be deduced: (1) Elevated *E. coli* MPN counts determined cluster C2a, which showed specificity for urban-affected sites, and did not directly correlate with other variables; (2) Strain diversity and temperature were directly correlated, which was also described in a previous study[7], and these two variables are considered to be major contributors determining the two main clusters C1 and C2; (3) BOD was also correlated with strain diversity and temperature, and high BOD values appeared to influence of cluster C1b1; (4) No correlations were observed between pH and the other variables; (5) The high frequency of phylogenetic group A strains appeared to be correlated with low values of strain diversity and temperature. This correlation was weak and was not as significantly correlated as was the relationship between strain diversity and temperature. The high frequency of occurrence of phylogenetic group A strains with low temperature is consistent with the increasing ratio of phylogenetic group A in the cold months as described above. Strains in phylogenetic group A are also considered to be a determining factor for the main clusters C1 and C2; (6) Phylogenetic group B1 strains were mostly present in high temperatures and showed a frequency distribution contrary to phylogenetic group A strains; (7) Phylogenetic group B2 strains did not directly correlate with any other variables and appeared to affect cluster C2b2; and (8) The high frequency of phylogenetic group D strains correlated with high temperatures and contributed to assignment of cluster C1a. Overall, temperature affected strain diversity and the occurrence of phylogenetic groups A, B1, and D. In contrast, phylogenetic group B2 was not affected by any other variables. However, the lack of any patterns in B2 strains and to a lesser extent D strains probably reflects their rarity.

### Correlation between distribution of *E. coli* phylogenetic groups and environmental factors

Redundancy analysis (RDA) was performed to determine correlations between prevalence of *E. coli* phylogenetic groups and environmental factors in surface water of the Yeongsan River basin. Five parameters such as BOD, temperature, pH, *E. coli* MPN, and strain diversity were used for the possible explanatory variables for constraining the occurrence of *E. coli* phylogenetic groups A and B1, which occupied more than 80% of total *E. coli* strains. The RDA explained 49.3% of the total variation in the phylogenetic groups A and B1.

**Figure 4:**
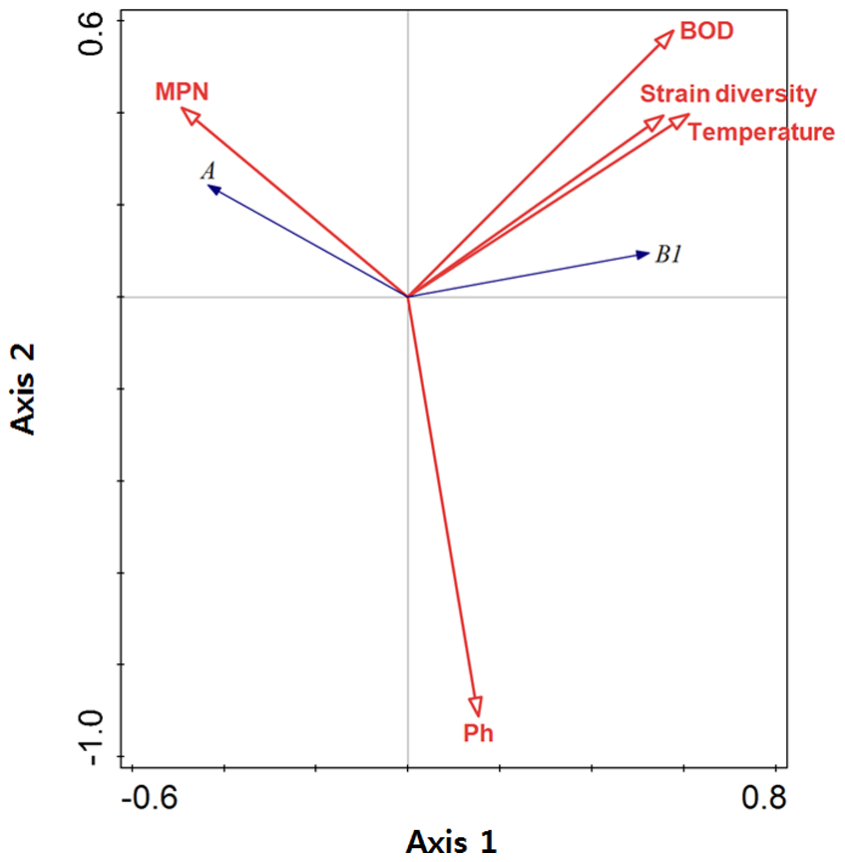
Ordination diagram from the redundancy analysis. The correlations of the environmental variables (explanatory variables) with the two phylognetic groups (response variables) can be predicted from angles between the arrows of the variables. The biplot projection of the phylogenetic group arrow tips onto the arrow of an environmental variable provides a more precise approximation.

The correlations between the two *E. coli* phylogenetic groups and the environmental data were shown in an ordination diagram (Fig. 4) showing the results which are similar to ones observed from the SOMs analyses. Temperature, strain diversity, and BOD were positively correlated to each other. The pH values were not likely to correlate with the other environmental factors and phylogenetic groups. Temperature, strain diversity, BOD and *E. coli* MPN were determined to be significant explanatory variables affecting the occurrences of the two *E. coli* phylogenetic groups (P<0.05) by using a forward selection method. High values of temperature, strain diversity, and BOD were correlated with high number of phylogenetic group B1, but with low number of phylogenetic group A. *E. coli* MPN, on the other hand, were positively correlated with phylogenetic group A, but negatively with B1. Furthermore, negative correlation was shown between phylogenetic group A and B1.

The correlation between *E. coli* MPN and phylogenetic group A may be affected by the fact that group A was most frequently found among total *E. coli* strains, suggesting that larger number of *E. coli* isolates provides more chance to obtain phylogenetic group A strains.

### Distribution of potential pathogenic *E. coli* strains among phylogenetic groups

**Figure 5:**
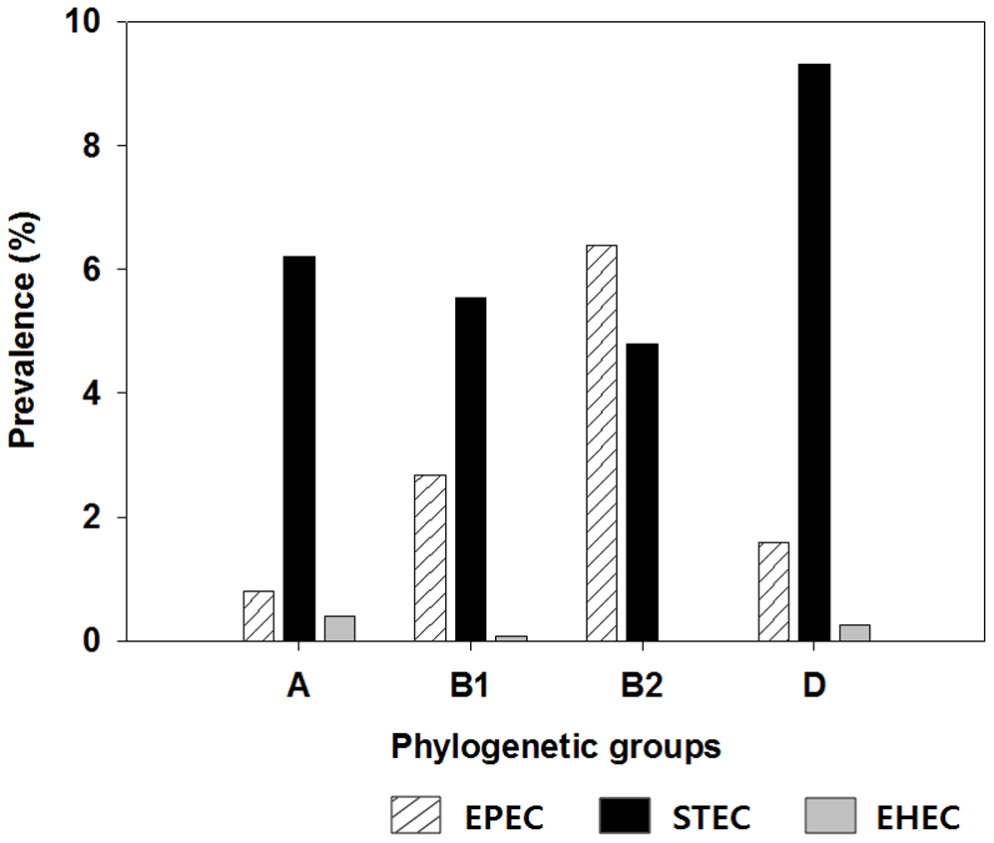
Prevalence of potential *E. coli* pathotypes EPEC, STEC, and EHEC among phylogenetic groups.

Based on previously accepted definitions of *E. coli* pathotypes [24], [44], *E. coli* strains carrying virulence genes were assigned to the pathotypes: *stx1, stx2 or stx1/stx2*, shiga toxigenic *E. coli* (STEC); *eaeA* with *stx1* or/and *stx2*, enterohemorrhagic *E. coli* (EHEC); *eaeA* without *stx1* or *stx2*, enteropathogenic *E. coli* (EPEC). Among the 3,480 *E. coli* strains examined, 217 (6.2%), 64(1.8%), and 9 (0.3%) were assigned to STEC, EPEC, and EHEC groups, respectively. Results in Figure 5 show that the greatest percentage of STEC strains was observed among phylogenetic group D (9.3%, 35 of 376). In contrast, phylogenetic group B2 included the greatest percentage of EPEC strains (6.4%, 12 of 188).

## Discussion

The separation of strains into phylogenetic groups A and B1 was also supported by genotypes defined by HFERP DNA fingerprint analyses as observed from MDS and jackknife analysis. MDS analyses indicated that although environmental factors such as temperature did affect the pattern of HFERP genotypes of *E. coli* strains [7], their phylogenetic groups that was correspondent with genotypic separation did not show any seasonal pattern of grouping (Fig. 1). This result is likely due to the fact that while phylogenetic grouping is based on indirect gross changes in enzyme mobility or stable genetic markers, rep-PCR technique directly detects changes in nucleotide base composition to identify possible rearrangements in bacterial genome structure. This suggests that the HFERP DNA fingerprinting method is more sensitive to genomic variations for adaption and plasticity in the environment than is phylogenetic grouping [31]. Furthermore, the seasonal specificity of *E. coli* HFERP genotypes, which was seen as a clustering of strains obtained from cold months, did not directly coincide with the distribution of *E. coli* phylogenetic groups as seen by the MDS analyses (Fig. 1). This also suggested that HFERP DNA fingerprint-defined genotypes of *E. coli* are more seasonally influenced by environmental factors, such as temperature than *E. coli* phylogenetic groups.

It is interesting that the SOMs analyses revealed that there was an inverse relationship between the occurrence of *E. coli* strains belonging to phylogenetic groups A and B1. Although the fact that phylogenetic groups A and B1 are inversely related to each other may simply reflect that they account for about 85% of the isolates, it is still remarkable since the inverse relation was correlated with the environmental factors. While *E. coli* strains belonging to phylogenetic group A was found to increase in the conditions such as low temperature and genotypical diversity, phylogenetic group B1 strains increased in the contrary conditions in the Yeongsan River basin, revealing that water temperature tends to be associated with the occurrence of specific *E. coli* phylogenetic groups. These correlations between *E. coli* phylogenetic groups and environmental factors were more clearly observed from the RDA. However, since phylogenetic groups were not strictly correlated with temperature as much as genotypic strain diversity did from the SOMs analyses, this suggests that other environmental factors may also cause differences between *E. coli* phylogenetic groups. In addition, the MDS analyses (Fig. 1) showed that phylogenetic group A was not comprised of strains obtained from only cold months, suggesting temperature is not only the factor affecting *E. coli* phylogeny.

Previously, Tenaillon et al. (2010) reported that the population structure of *E. coli* is predominantly clonal in spite of the occurrence of recombination events, allowing the delineation of major phylogenetic groups [14]. Based on the results reported here, our data suggest that *E. coli* phylogenetic groups are adaptable and genotypically-influenced by changes in environmental conditions. This conclusion is in large part supported by our observation that phylogenetic group A strains appear to be more adaptable to low temperature than the other groups examined in this study. While several previous studies already have reported different *E. coli* ecological structures depending on their origins, few studies described the specific environmental factors affecting *E. coli* phylogenetic groups in river water.

As described previously [7], no site-specificity of *E. coli* genotypes and phylogenetic groups was observed in this study as well. However, the representativeness of the *E. coli* strains for each surface water sample is quite variable because of the same number of the strains obtained from each sample, irrespective of how much total population of *E. coli* is in there. Particularly the representativeness of the *E. coli* isolates in urban sites showing generally high MPN values is likely to be lower than the other sites. Thus, it would be improper to discuss on site-specificity of the results in this study.

Although the total number of *E. coli* strains belonging to phylogenetic groups B2 and D occurred less frequently than ones belonging to the other phylogenetic groups, the percentage of potential *E. coli* pathotypes comprising EPEC and STEC strains were highest among members of groups B2 and D. This result is consistent with previous studies that phylogenetic groups A and B1 members were often comprised of commensal stains and that phylogenetic groups B2 and D were observed to contain more virulent strains than other groups [18], [45]–[47]. Thus, the previously reported relationship between phylogeny and virulence of *E. coli* strains can similarly be applied to the *E. coli* population existing in surface waters of the Yeongsan River basin. However, it should be noted that typical strains of EHEC O157:H7 possessing no β-d-glucuronidase were excluded from the results since *E. coli* strains examined in this study were initially obtained by using mTEC agar plates which has selectivity for the bacterial species based on β-d-glucuronidase activity [48].

In conclusion, we show that there is a relationship between genotypic groupings of *E. coli* based on their HFERP DNA fingerprints and strains in phylogenetic groups A and B1. Moreover, we report here that the SOMs and RDA analyses indicate that the distributional changes of these groups are affected by the environmental factors. The compositional differences of phylogenetic groups among *E. coli* populations in different environments may be caused by differences in adaptability and plasticity of *E. coli* strains belonging to each phylogenetic group. Environmentally-adapted genotypes and changes in phylogenetic groups according to specific environmental conditions probably are not related to temporal deposition of fecal pollutant since they were observed regardless of sampling locations. The genetic bases for these changes are unknown, and further investigations of genomic DNA sequence variation among the *E. coli* strains examined in the current study will likely provide in-depth information about the genotypic changes imparted by environmental conditions of the river water. Moreover, it would be worthwhile to perform a further repetitive monitoring of *E. coli* populations in the environment to confirm the reproducibility of the study results during a longer sampling period since a river environment is not a static ecosystem.

## Supporting Information

Table S1 **Raw data of water parameters and** ***E. coli*** **phylogenetic groups for each surface water sample.**
(XLS)
